# MiR-21-5p enhances differentiation and mitigates oleic acid-induced lipid droplet accumulation in C2C12 myoblasts by targeting FBXO11

**DOI:** 10.5713/ab.24.0665

**Published:** 2025-02-27

**Authors:** Yi Li, Jiayuan Fang, Yingying Jiao, Qinchuan Lv, Xingyu Xiao, Shuo Zheng, Xi Chen, Jie Song, Xunming Zhang, Libo Zhang, Ze Ma, Changhong Li, Linlin Hao

**Affiliations:** 1 College of Animal Science, Jilin University, Changchun, China; 2 College of Life Science, Baicheng Normal University, Baicheng, China

**Keywords:** C2C12 Myoblast, Differentiation, FBXO11, Intramuscular Lipid Deposition, miR-21-5p, Proliferation

## Abstract

**Objective:**

This study aimed to investigate the role of miRNA 21-5p in regulating the differentiation of C2C12 myoblasts and intramuscular lipid droplets accumulation in myotubes.

**Methods:**

The role of miR-21-5p in the proliferation and differentiation of myofibroblasts and intracellular lipid accumulation was analyzed using bioinformatics, CCK-8 assay, quantitative real-time polymerase chain reaction, immunoblotting, immunofluorescence staining, and Oil Red O staining.

**Results:**

The analysis of porcine BodyMap transcriptome data revealed differential expression of miRNA 21-5p in skeletal muscle and adipose tissue. Bioinformatics analysis combined with a dual-luciferase reporter assay demonstrated that FBXO11 serves as a direct target of miR-21-5p. Transfection experiments involving a miR-21-5p mimic, miR-21-5p inhibitor, and si-FBXO11 in C2C12 cells showed that overexpression of miR-21-5p or silencing of FBXO11 significantly enhanced the proliferation of C2C12 cells, upregulated myogenesis-related factors, and promoted myotube formation. Furthermore, oleic acid-induced lipid accumulation in myotubes was suppressed, accompanied by reduced expression of adipogenesis-related genes. Conversely, inhibition of miR-21-5p expression produced opposite effects.

**Conclusion:**

These findings indicate that miR-21-5p promotes proliferation and differentiation while inhibiting intramyocellular lipid deposition by targeting the 3′-untranslated region of FBXO11 in myogenic cell. The results suggest that miR-21-5p could serve as a potential miRNA biomarker for regulating intramuscular adipogenesis.

## INTRODUCTION

Meat quality is closely associated with skeletal muscle development and intramuscular fat (IMF) deposition, influenced by factors including genetics [1,2]. IMF, formed through the accumulation of triglycerides and fatty acids within skeletal muscle, is stored in muscle fibers as intramyocellular lipids in the form of non-cellular lipid droplets [3,4]. A moderate increase in IMF content enhances the flavor, tenderness, and juiciness of meat [5]. Numerous miRNAs identified via RNA-seq have been implicated in the regulation of IMF content, though their specific roles and mechanisms require further exploration [6].

MiRNAs are a highly conserved class of endogenous non-coding small RNAs that modulate mRNA degradation or translational inhibition by targeting the 3′-untranslated region (3′UTR) [7,8]. Studies have shown that miRNAs are involved in myoblast proliferation and differentiation, regulate lipid droplet accumulation, and play critical roles in skeletal muscle growth and IMF deposition [6,9–12]. For instance, miR-34a regulates lipid droplet accumulation in C2C12 cells by targeting lymphocyte enhancer factor-1 [13]. Similarly, miR-324-5p significantly inhibits C2C12 myoblast differentiation through long non-coding Dum (lncDum) and peptidase M20 domain containing 1 (Pm20d1), while promoting oleate-induced lipid accumulation in myoblasts [14]. Myostatin has been reported to activate miR-124-3p, suppressing glucocorticoid receptor expression and indirectly affecting IMF deposition [15]. MiR-21, a highly conserved and abundant miRNA in porcine skeletal muscle and IMF, is suspected to play a pivotal role in regulating porcine IMF deposition through its target genes.

Previous studies have demonstrated that miR-21-5p participates in porcine skeletal muscle development by modulating the PI3K/AKT/mTOR signaling pathway [16]. Additionally, overexpression of miR-21-5p has been shown to inhibit lipid accumulation in H9C2 cells [17] and significantly suppress stearic acid -induced intracellular lipid accumulation in mouse hepatocellular carcinoma cells by downregulating fatty acid binding protein-7 (FABP7) expression [18]. Transcriptomic data indicate that miR-21-5p is highly expressed in porcine intramuscular adipocytes, with its expression declining during differentiation [19]. This suggests a significant role for miR-21-5p in skeletal muscle development and IMF deposition, although its regulatory mechanisms remain unexplored.

Bioinformatics predictions identified *FBXO11* as a direct target gene of miR-21-5p. *FBXO11*, a member of the F-box protein family [20], has been primarily studied for its role in cancer development [21,22]. However, a related family member, *FBXO32*, is known to play a crucial role in muscle atrophy [23]. Interestingly, *FBXO11* has been reported to exhibit differential expression in normal diaphragm muscle versus x-linked muscular dystrophy (MDX) diaphragm and in the IMF of various chicken breeds [24,25]. These findings imply that *FBXO11*, as a target of miR-21-5p, may contribute to skeletal muscle development and the regulation of intramuscular lipid deposition.

Mouse C2C12 cells, which can be induced to differentiate into myoblasts under *in vitro* conditions, are extensively utilized in research on skeletal muscle development. Previous studies have demonstrated that oleic acid induction enhances triglyceride accumulation in myotubes derived from C2C12 cells [26,27]. In the present study, miR-21-5p and *FBXO11* were found to exhibit differential expression in the longissimus dorsi muscle (LDM), IMF, and skeletal muscle satellite cells from Landrace (lean pig; LW) and Bama minipigs (fatty pig; BM). Furthermore, miR-21-5p was observed to promote the proliferation and differentiation of C2C12 cells while inhibiting the accumulation of intramyocellular lipid droplets by targeting *FBXO11*. These regulatory effects may explain the observed differences in lean muscle percentage and IMF content between these pig breeds.

## MATERIALS AND METHODS

### Animal experiments and cells

Samples of the LDM and IMF were collected from three male LW and three BM at 7 days of age. Porcine skeletal muscle satellite cells (PSCs) were isolated from porcine leg muscle tissues using the method described by Li et al [28]. All animal experiments received approval from the Experimental Animal Center of Jilin University and were conducted in accordance with the ethical guidelines outlined in the Guide for Ethical Use of Animals provided by the Animal Welfare and Research Ethics Committee of Jilin University (approval no. KT202003090).

### Cell culture and treatments

The 3T3-L1, C2C12, and 293T cell lines used in the experiments were kindly provided by Prof. Hongsheng Ouyang from the College of Animal Science at Jilin University. These cell lines were cultured in a CO_2_ incubator at 37°C using Dulbecco’s Modified Essential Medium (DMEM; Gibco, Grand Island, NY, USA) supplemented with penicillin, streptomycin (Gibco), and 10% fetal bovine serum (FBS; Gibco), referred to as M1 medium. PSCs were maintained in DMEM/F-12 (Sigma-Aldrich, St. Louis, MO, USA) supplemented with 20% FBS, 0.5% penicillin/streptomycin, and 0.5% chick embryo extract in an incubator with 5% CO_2_ at 37°C.

For adipogenic differentiation, 3T3-L1 cells were cultured until 90% confluence (day 0), after which the initial differentiation medium (stage I) was replaced with M1 medium containing 10 μg/mL insulin (Yuanye Bio-Technology, Shanghai, China), 1 μM dexamethasone (Sigma-Aldrich), 200 μM indomethacin (Yuanye Bio-Technology), and 0.5 mM IBMX (Sigma-Aldrich). On day 2, the medium was changed to DMEM with 10% FBS and 10 μg/mL insulin. From day 9 onwards, insulin was excluded, and cells were maintained in M1 medium with medium replacement every 2 days.

For myogenic differentiation, C2C12 cells were cultured in DMEM supplemented with 2% horse serum (Solarbio, Beijing, China) once they reached 90% confluence. The culture medium was replaced every 48 h. After 6 days of differentiation, visible myotubes were formed. These cells were then treated with DMEM supplemented with 0.5% bovine serum albumin (Beyotime Biotechnology, Shanghai, China) and 500 μM oleic acid for 24 h to induce lipid accumulation within the myotubes.

### Plasmid construction and cell transfection

For the psiCHECK-2 dual-luciferase reporter vector, the 3′UTR fragment of *FBXO11*, which contains the miR-21-5p binding site, was cloned into the psiCHECK-2 vector. These fragments, designated *FBXO11*-WT and *FBXO11*-Mut, were provided by Sangon Biotech (Shanghai, China). RNA oligonucleotides used in this study, including miR-21-5p mimic, mimic-negative control (NC), miR-21-5p inhibitor, inhibitor-NC, si-FBXO11, and si-NC, were sourced from RiboBio Co., Ltd. (Guangzhou, China). Cells were seeded in six-well plates and cultured for 12 h prior to transfection with the mimics and their respective controls. Transfection of oligonucleotides and plasmids was conducted using LipoPlus Reagent (SageCreation, Beijing, China) following the manufacturer’s protocol, with all experiments performed in at least three independent replicates.

### Dual luciferase reporter assay

For luciferase reporter analysis, 293T cells were seeded into 96-well plates and cultured until reaching 80% confluence. Cells were then co-transfected with either *FBXO11*-WT or *FBXO11*-Mut constructs alongside ssc-miR-21-5p mimics or controls. After 48 h of transfection, firefly, and Renilla luciferase activities were quantified using a Fluorescence/Multi-Detection Microplate Reader (BioTek, Winooski, VT, USA) in conjunction with a Dual-Luciferase Reporter Gene Assay kit II (Beyotime Biotechnology). The luciferase activity was normalized to the corresponding firefly luciferase activity to ensure consistency across samples.

### Immunofluorescence staining

C2C12 cells were cultured in 24-well plates lined with cell slides, then transfected with ssc-miR-21-5p mimic, mimic-NC, ssc-miR-21-5p inhibitor, inhibitor-NC, si-FBXO11, or siRNA-NC. These cells were subsequently induced for myogenic differentiation over 6 days. After the differentiation period, the medium was removed, and cells were rinsed three times with PBS before being fixed with 4% paraformaldehyde for 20 min at room temperature. Following fixation, cells were washed thrice with PBS and permeabilized using 0.1% Triton X-100 for 30 min. The cells were then blocked with 10% FBS for 30 min and incubated overnight with anti-Myosin Heavy Chain (1:200; R&D Systems, Minneapolis, MN, USA). After three 5-min PBS washes, the cells were incubated with secondary antibodies (FITC-labeled Goat Anti-Mouse IgG (H+L), 1:100; Bioworld, St. Louis Park, MN, USA) for 2 h at room temperature, followed by additional PBS washes. Nuclei were stained with DAPI (Beyotime Biotechnology) for 5 min. Images were captured using an inverted fluorescence microscope (Nikon, Tokyo, Japan), and the data were analyzed with ImageJ software (National Institutes of Health, Bethesda, MD, USA).

### CCK-8 assay

C2C12 cells were seeded into 96-well plates at a density of 2000 cells per well and transfected with miR-21-5p mimic, mimic-NC, miR-21-5p inhibitor, inhibitor-NC, si-FBXO11, or siRNA-NC. Cell proliferation was evaluated at 0, 24, 48, 72, and 96 h using a Cell Counting Kit-8 (CCK-8) (SAINT-BIO, Shanghai, China) according to the manufacturer’s instructions.

### Oil Red O staining

Lipid droplet formation in cultured cells was assessed using Oil Red O staining. Differentiated C2C12 myotubes were treated with oleic acid for 24 h and subsequently stained. Cells were first rinsed with PBS and fixed with 4% paraformaldehyde (Beyotime Biotechnology) for 20 min. Following fixation, the cells were briefly washed in 60% isopropanol and stained with Oil Red O working solution (Solarbio) for 20 min. Stained cells were washed with 60% isopropanol for 30 s and rinsed 3 to 5 times with PBS. To quantify lipid content, Oil Red O was extracted using isopropanol, and the optical density was measured at 510 nm. After washing with Oil Red O buffer for 1 min, cells were covered with PBS and observed under a microscope (Nikon).

### Quantitative real-time polymerase chain reaction

Total RNA was isolated from tissues or cells using TRIzol reagent (Takara, Kusatsu, Japan). For mRNA, cDNA synthesis was performed using the TransScript Uni All-in-One First-Strand cDNA Synthesis SuperMix for qPCR (TransGen Biotech, Beijing, China). miRNA reverse transcription was conducted using the miRNA First Strand cDNA Synthesis Tailing Reaction Kit (Sangon Biotech). Quantitative real-time polymerase chain reaction (qRT-PCR) was performed using the TransStart Green qPCR SuperMix (TransGen Biotech) on an ABI PRISM 7900HT thermocycler (Applied Biosystems, Foster City, CA, USA). The comparative Ct (2^−ΔΔCt^) method was employed for data analysis. All reactions were performed in triplicate. For normalization, GAPDH served as the internal reference for mRNA analysis, whereas U6 was used as the internal control for miRNA quantification. Primers used in the qRT-PCR experiments are detailed in Supplement 1.

### Western blot analysis

The procedure for Western Blot analysis was adapted slightly from previously established protocols. Primary antibodies utilized in this study included FBXO11 (1:500; Santa Cruz Biotechnology, CA, USA), MyoD (1:500; Santa Cruz Biotechnology), and GAPDH (Bioworld, 1:10,000). The secondary antibody used was an horseradish peroxidase (HRP)-conjugated goat anti-mouse IgG antibody (Bioworld, 1:10,000).

### Statistical analysis

All experiments were performed in three independent replicates, and the results are presented as mean±standard error of the mean. Statistical analysis was conducted using GraphPad Prism Software (Version 8; La Jolla, CA, USA). Comparisons between two groups were made using Student’s t-test to determine statistical significance. A p-value of <0.05 was considered to be statistically significant (* p<0.05, ** p<0.01, *** p< 0.001).

## RESULTS

### The role of miR-21-5p in skeletal muscle development and intramuscular fat accumulation

**Figure 1:**
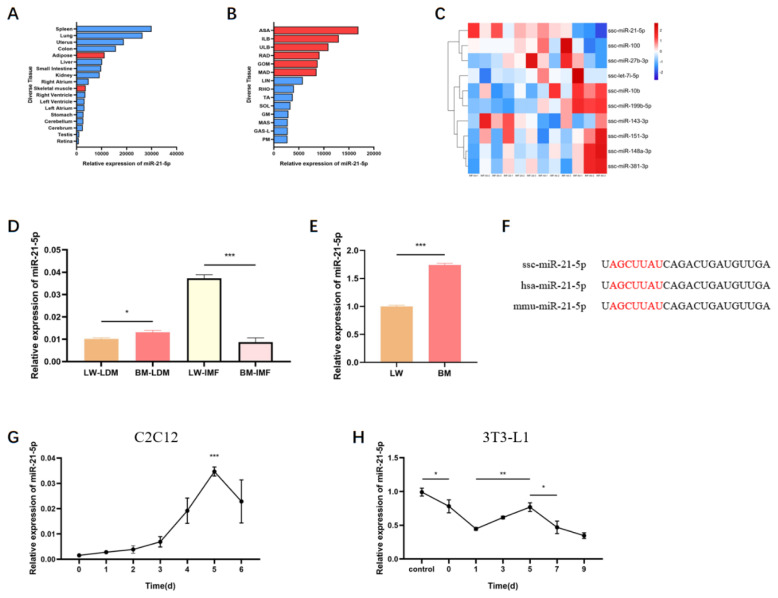
The role of miR-21-5p in skeletal muscle development and intramuscular fat accumulation. (A) Expression of miR-21-5p in different tissues of pigs, (B) expression of miR-21-5p in different skeletal muscles and adipose tissues of pig, (C) heat map visualizing the expression profile of miRNAs during porcine intramuscular adipocyte differentiation, (D) expression levels of miR-21-5p in LDM and IMF of LW and BM, (E) expression levels of miR-21-5p in PSCs, (F) conservation analysis of miR-21-5p, (G-H) temporal expression of miR-21-5p during differentiation of C2C12 cells and 3T3-L1 cells. LW, Landrace pigs; LDM, longissimus dorsi muscle; BM, Bama minipigs; IMF, intramuscular fat; PSCs, porcine skeletal muscle satellite cells. * p<0.05, ** p<0.01, *** p<0.001.

The pig BodyMap transcriptome dataset (GEO: GSE162147) [29] demonstrated that miR-21-5p was abundantly and differentially expressed across various porcine tissues, including skeletal muscle and adipose tissues at distinct anatomical sites (Figures 1A, 1B). Additionally, analysis of miR-21-5p expression during the differentiation of porcine intramuscular adipocytes (GEO: GSE193608) [19] revealed significant downregulation (Figure 1C). The expression of miR-21-5p in LDM and IMF and PSCs of two pig breeds: LW and BM, was compared. The results showed that miR-21-5p was differentially expression in these tissues and cells between the breeds (Figures 1D, 1E). The mature sequence of miR-21-5p is highly conserved among mammals, with notable sequence similarity across human, mouse, and pig species (Figure 1F). To further examine its expression patterns during differentiation, C2C12 cells and 3T3-L1 cells were used as models. In C2C12 cells, miR-21-5p expression progressively increased during myogenic differentiation. Conversely, in 3T3-L1 cells, miR-21-5p exhibited a three-phase expression change with an overall decreasing trend, mirroring the pattern observed in porcine intramuscular adipocytes (Figures 1G, 1H). These results highlighted that miR-21-5p shows contrasting expression trends during myogenic and adipogenic differentiation, suggesting distinct roles in regulating skeletal muscle development and IMF deposition.

### MiR-21-5p promotes the proliferation of C2C12

**Figure 2:**
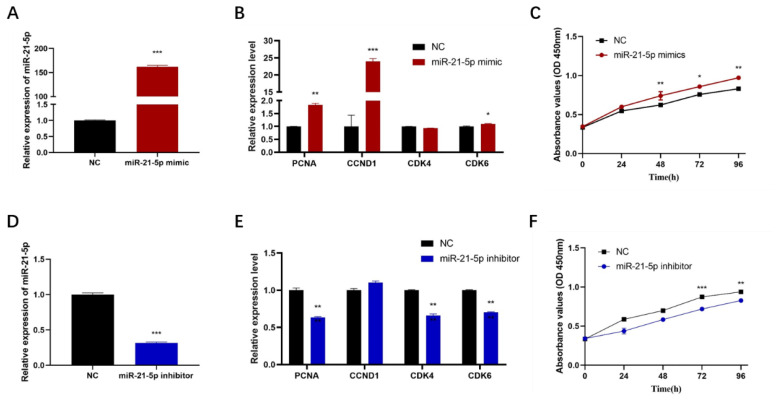
Effect of miR-21-5p on C2C12 cell proliferation. (A, D). Transfection efficiency of miR-21-5p inhibitor and miR-21-5p mimic, (B, E). C2C12 cell cycle-related factor mRNA expression levels after transfection, (C, F) Effect of miR-21-5p on proliferation ability of C2C12 by CCK8 assay. The data are expressed as mean±SD (n = 3/group. * p<0.05, ** p<0.01, and *** p<0.001). NC, negative control; SD, standard deviation.

Mouse C2C12 cells were utilized as a model system because primary porcine cells exhibit low transfection efficiency, and no porcine cell lines are currently available. Transfected C2C12 cells with synthetic mimics or inhibitors of miR-21-5p were analyzed for miR-21-5p expression and several cell cycle-related genes using RT-qPCR. Additionally, CCK-8 assays were conducted to evaluate the proliferation of C2C12 cells at various time points post-transfection. The results demonstrated that transfection with miR-21-5p mimics or inhibitors significantly altered miR-21-5p expression levels (Figures 2A, 2D). Overexpression of miR-21-5p markedly increased the mRNA levels of PCNA, CCND1, and CDK6, thereby promoting C2C12 cell proliferation (Figures 2B, 2C). Conversely, knockdown of miR-21-5p reduced the mRNA expression levels of PCNA, CDK4, and CDK6, resulting in inhibited proliferation of C2C12 cells (Figures 2E, 2F). These findings indicate that miR-21-5p acts as a positive regulator of myoblast proliferation *in vitro*.

### MiR-21-5p promotes C2C12 myoblast differentiation

**Figure 3:**
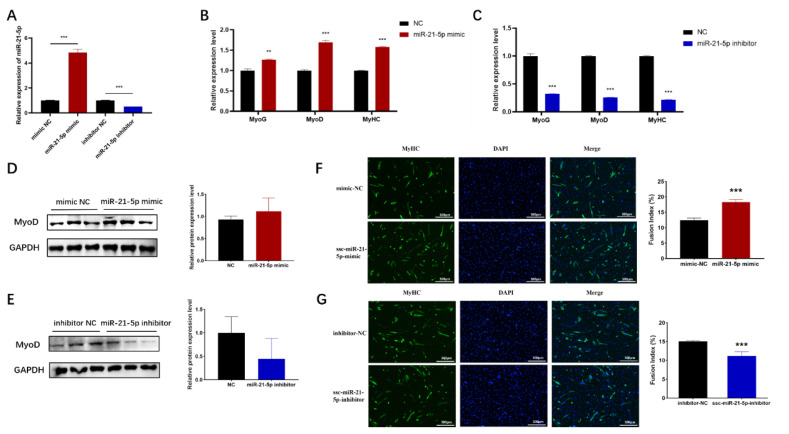
Effect of miR-21-5p on myogenic differentiation in C2C12 cell. (A) miR-21-5p expression levels after overexpression or knockdown, (B–C) Myogenesis-related factor mRNA expression levels, (D–E) MyoD protein expression levels, (F–G) MyHC immunofluorescence staining analysis and cell fusion index of myotube formation. The data are expressed as mean±SD (n = 3/group. * p<0.05, ** p<0.01, and *** p<0.001). NC, negative control; SD, standard deviation.

To investigate the role of miR-21-5p in myoblastic differentiation, C2C12 cells were transfected with miR-21-5p mimics or inhibitors and induced to differentiate in a low-serum medium. After 6 days, myotube formation was evaluated using immunofluorescence staining for myosin heavy chain (MyHC), while miR-21-5p levels and the expression of myogenesis-related genes were analyzed by RT-qPCR and western blotting. Overexpression or inhibition of miR-21-5p resulted in significant upregulation or downregulation of miR-21-5p expression, respectively (Figure 3A). Compared to NC, miR-21-5p overexpression enhanced myotube formation, whereas inhibition impaired it (Figures 3F, 3G). Additionally, miR-21-5p overexpression elevated the mRNA levels of MyoD, MyoG, and MyHC (Figure 3B), as well as the protein expression level of MyoD tended to increase (Figure 3D), while its inhibition reduced these markers (Figures 3C, 3E). These findings suggest that miR-21-5p positively regulates myogenic differentiation in C2C12 cells by modulating the expression of myogenesis-related genes.

### MiR-21-5p decreases oleic acid-induced lipid deposition in C2C12 myoblasts

**Figure 4:**
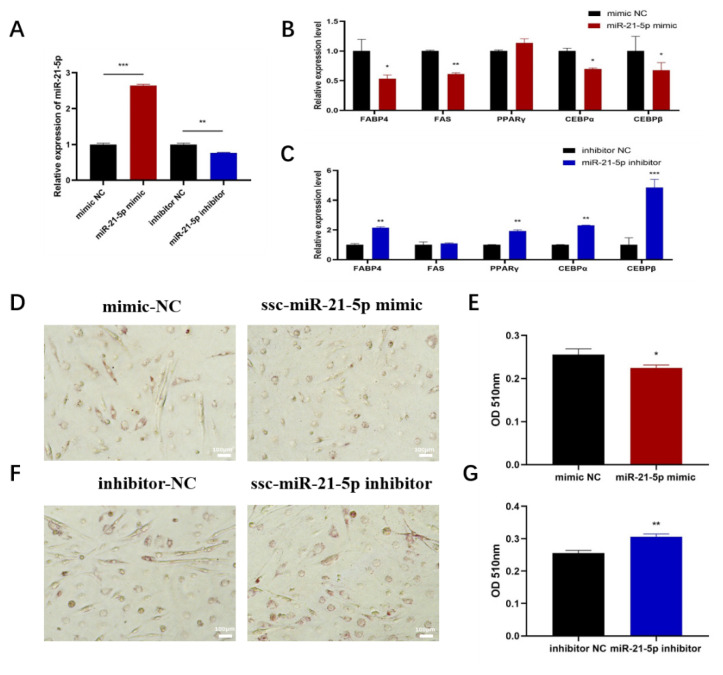
Effect of miR-21-5p on oleic acid-induced lipid deposition in C2C12 myoblasts. (A) Transfection efficiency of miR-21-5p inhibitor and miR-21-5p mimic, (B, C) Effect of transfection of miR-21-5p inhibitor and miR-21-5p mimic on the expression level of mRNA of lipogenic related factors, (D, F) Oil red O staining for lipids in C2C12 myoblasts, (E, G) Oil red O-stained cells were extracted with isopropyl alcohol for assessment of intracellular triglyceride concentrations. The data are expressed as mean±SD (n = 3/group. * p<0.05, ** p<0.01, and *** p<0.001). NC, negative control; OD, optical density; SD, standard deviation.

Oleic acid has been shown to influence the lipid deposition in C2C12-derived myotubes. The effect of ssc-miR-21-5p on oleic acid-induced lipid accumulation in differentiated C2C12 cells was examined. At day 6 of differentiation, C2C12 cells were transfected with miR-21-5p mimics or inhibitors and exposed to 500 μmol/L oleic acid. Lipid accumulation was assessed using oil red O staining, and the expression of lipogenic factors was measured by RT-qPCR. Transfection with miR-21-5p mimics or inhibitors altered the expression of miR-21-5p when compared with the NC (Figure 4A). The results of oil red O staining showed that overexpression of miR-21-5p inhibited intracellular lipid deposition in myotubes and significantly decreased intracellular triglyceride concentrations (Figures 4D, 4E). However, the amount of lipid and the intracellular triglyceride concentration was increased after knockdown of miR-21-5p (Figures 4F, 4G). RT-qPCR analysis revealed that miR-21-5p overexpression significantly downregulated the mRNA levels of FABP4, FAS, CEBPα, and CEBPβ (Figure 4B), while its inhibition upregulated FABP4, PPARγ, CEBPα, and CEBPβ (Figure 4C). These findings demonstrate that miR-21-5p inhibits oleic acid-induced lipid deposition in C2C12 myotubes.

### *FBXO11* is the direct target gene of miR-21-5p

**Figure 5:**
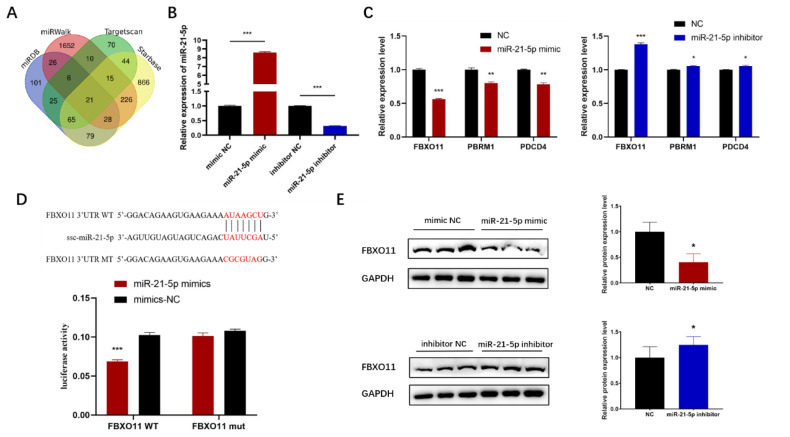
FBXO11 is a direct target gene of miR-21-5p. (A) Four miRNA target gene prediction tools to predict potential miR-21-5p target genes, (B) transfection efficiency of miR-21-5p inhibitor and miR-21-5p mimic, (C) expression of target gene mRNAs in porcine skeletal muscle satellite cells transfected with miR-21-5p inhibitor or mimic, (D) miR-21-5p binding site in the FBXO11 mRNA 3’UTR and Dual-luciferase reporter assay of the co-transfection of wild-type or mutant FBXO11 3′UTR with miR-21-5p mimics or mimics-NC in 293T cells; (E) effect of miR-21-5p on the protein expression level of FBXO11. The data are expressed as mean±SD (n = 3/group. * p<0.05, ** p<0.01, and *** p<0.001). NC, negative control; SD, standard deviation.

MiR-21-5p plays a critical role in the proliferation and differentiation of C2C12 cells as well as lipid deposition; however, its underlying regulatory mechanism remains unclear. To investigate this mechanism, potential target genes of miR-21-5p were identified using four miRNA target gene prediction tools: TargetScan, StarBase, miRDB, and miRWalk. The intersection of these prediction results was analyzed (Figure 5A). Homology analyses of porcine and mouse sequences were conducted for regions before and after 100 bp of their 3′UTR binding sites, resulting in the selection of three candidate target genes with high homology and binding sites within 300 bp: *PBRM1*, *PDCD4*, and *FBXO11*. To evaluate the targeting potential of these genes, miR-21-5p mimics and inhibitors were transfected into PSCs (Figure 5B). Among the candidate genes, *FBXO11* exhibited the most significant downregulation or upregulation compared to NC (Figure 5C). A dual luciferase reporter gene assay was conducted to confirm whether *FBXO11* is a direct target gene of miR-21-5p (Figure 5D). Additionally, the expression of *FBXO11* was assessed by overexpressing or knocking down miR-21-5p in C2C12 cells (Figures 2A, 2D). Western blot analysis revealed that miR-21-5p overexpression significantly suppressed *FBXO11* expression, while knockdown of miR-21-5p enhanced *FBXO11* expression (Figure 5E). These findings collectively confirm the targeting relationship between miR-21-5p and *FBXO11*.

### *FBXO11* inhibits C2C12 proliferation and myoblast differentiation

**Figure 6:**
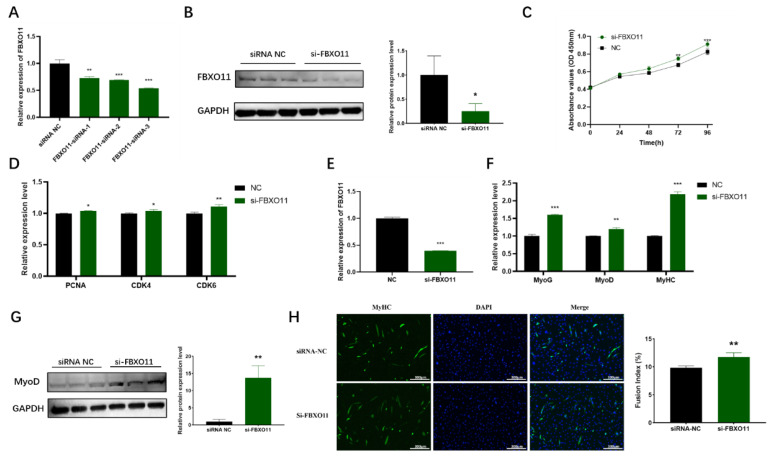
Effect of FBXO11 on C2C12 cell proliferation and myogenic differentiation. (A) FBXO11 mRNA expression level in C2C12 cells after transfection with three types of si-FBXO11, (B) FBXO11 protein expression level in C2C12 cells after transfection with si-FBXO11-3, (C) CCK-8 assay after the transfection of si-FBXO11 into C2C12, (D) mRNA expression levels of cell cycle marker genes, (E) transfection efficiency of si-FBXO11, (F) myogenesis-related factor mRNA expression levels, (G) MyoD protein expression levels, (H) MyHC immunofluorescence staining analysis and cell fusion index of myotube formation. The data are expressed as mean±SD (n = 3/group. * p<0.05, ** p<0.01, and *** p<0.001). NC, negative control; SD, standard deviation.

Previous studies have suggested that *FBXO11* plays a pivotal role in the development of skeletal muscle [24,25]. To investigate the effect of *FBXO11* on C2C12 cell proliferation, three distinct si-FBXO11 constructs were transfected into C2C12 cells. Among these, si-FBXO11-3 resulted in the most pronounced reduction in FBXO11 mRNA and protein expression (Figures 6A, 6B). Following transfection with si-FBXO11-3, a CCK-8 assay was performed to evaluate cell proliferation at different time points, while RT-qPCR was used to analyze the expression of cell cycle-related genes. The results indicated that *FBXO11* silencing significantly upregulated the mRNA expression of *PCNA*, *CDK4*, and *CDK6*, thereby enhancing C2C12 cell proliferation (Figures 6C, 6D). To explore the role of *FBXO11* in myogenic differentiation, C2C12 cells transfected with si-FBXO11 or siRNA-NC were induced to differentiate in a low-serum medium for six days. Transfection efficiency and myotube formation were assessed using MyHC immunofluorescence staining, while RT-qPCR and western blotting were used to evaluate the expression of myogenesis-related genes. *FBXO11* silencing significantly reduced its expression levels (Figure 6E) and enhanced myotube formation relative to the control (Figure 6H). Moreover, *FBXO11* inhibition markedly increased the mRNA levels of *MyoG*, *MyoD*, and *MyH*C (Figure 6F), as well as the protein level of MyoD (Figure 6G). These results demonstrate that *FBXO11* suppression promotes C2C12 proliferation and myogenic differentiation *in vitro*. Interestingly, these effects contrast with the role of miR-21-5p in regulating C2C12 cell proliferation and differentiation, suggesting that miR-21-5p exerts its influence on these processes by targeting *FBXO11*.

### Silencing of *FBXO11* decreases oleic acid-induced lipid deposition in C2C12 cells

**Figure 7:**
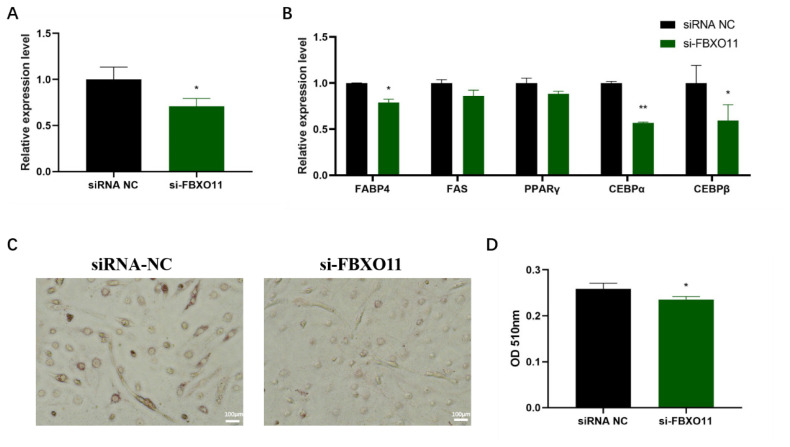
Effect of FBXO11 on lipid deposition in C2C12 myoblasts. (A) Transfection efficiency of si-FBXO11, (B) Effect of transfection of si-FBXO11 on the expression level of mRNA of lipogenic related factors, (C) Oil red O staining of lipids in C2C12 myotubes, (D) Isopropanol extraction of oil red O stained cells to assess intracellular triglyceride concentrations. The data are expressed as mean±SD (n = 3/group. * p<0.05, ** p<0.01). NC, negative control; OD, optical density; SD, standard deviation.

To investigate whether miR-21-5p inhibits lipid deposition in myocytes by targeting *FBXO11* and to validate the role of *FBXO11* in lipid deposition in C2C12 myocytes induced by oleic acid, si-FBXO11, and siRNA-NC were transfected into C2C12 cells undergoing myogenic differentiation for six days, followed by oleic acid induction. Transfection efficiency was assessed, and Oil Red O staining was employed to evaluate lipid deposition, alongside the measurement of mRNA expression levels of adipogenesis-related factors. si-FBXO11 effectively downregulated FBXO11 mRNA expression compared to NC (Figure 7A). Oil Red O staining revealed that *FBXO11* inhibition reduced intracellular lipid accumulation in myotubes and significantly lowered intracellular triglyceride levels (Figures 7C, 7D). Moreover, RT-qPCR analysis demonstrated that suppressing *FBXO11* expression markedly decreased the mRNA levels of adipogenesis-related genes, including FABP4, CEBPα, and CEBPβ (Figure 7B). These results indicate that *FBXO11* silencing effectively inhibits lipid accumulation induced by oleic acid in myotubes derived from C2C12 myoblasts.

## DISCUSSION

Skeletal myogenesis is a complex biological process [30]. As a small regulatory non-coding RNA, miRNA plays a significant role in skeletal muscle development and intramuscular lipid deposition [31,32]. While prior research has extensively studied the role of miR-21-5p in vascular smooth muscle, cardiac muscle, and avian skeletal muscle, its involvement in mammalian skeletal muscle formation and lipid deposition remains largely unexplored [8,33,34]. Bioinformatics analysis of available transcriptomic data, as reported by Zhang et al [19] and Jin et al [29], revealed that miR-21-5p exhibits high expression levels across various pig tissues, with notably greater expression in adipose tissues than in skeletal muscle tissues. Furthermore, its expression decreases during adipocyte differentiation in porcine intramuscular muscle (Figures 1A–1C). In this study, a similar expression trend for miR-21-5p was observed during the differentiation of C2C12 and 3T3-L1 cells, aligning with transcriptome analysis findings (Figures 1E, 1F). Additionally, distinct regulatory patterns emerged during muscle development and intramuscular lipid deposition (Figures 1D–1H). These observations imply a potential role for miR-21-5p in skeletal muscle development and intramuscular lipid formation; however, further evidence is required to substantiate this hypothesis.

To further validate the mechanism underlying miR-21-5p activity, target genes were identified using online prediction tools, and their interactions were confirmed through luciferase reporter assays. The results demonstrated that miR-21-5p significantly reduced luciferase activity in wild-type *FBXO11* 3′UTR constructs, whereas no significant effect was observed in mutant *FBXO11* 3′UTR constructs (Figure 5). F-box proteins, categorized into FBXL, FBXW, and FBXO groups based on their C-terminal secondary structure, share conserved F-box motifs at their N-termini [35,36]. Previous research has highlighted differential expression of *FBXO11* in normal and MDX diaphragm muscles and in IMF across diverse chicken breeds [24,25]. Moreover, *FBXO32*, another member of the FBXO group, plays a critical role in muscle atrophy [37,38]. These findings suggest that the miR-21-5p-*FBXO11* interaction represents a pivotal miRNA-mRNA regulatory axis affecting skeletal muscle development and intramuscular lipid deposition, particularly in pigs with varying meat quality.

Proliferation and differentiation of myoblasts are essential for skeletal myogenesis, and C2C12, a murine-derived myogenic cell line, serves as a standard model for studying skeletal muscle development and lipid deposition [14,39]. In this study, C2C12 cells were employed to explore the role of the miR-21-5p-*FBXO11* axis in these processes. Transfection experiments involving miR-21-5p mimics, inhibitors, and si-FBXO11 during cell proliferation and differentiation revealed that overexpression of miR-21-5p or *FBXO11* silencing significantly enhanced C2C12 proliferation and upregulated cell-cycle regulatory factors [40,41]. Furthermore, these conditions increased the expression of myogenic marker genes (MyoD, MyHC, MyoG) [42] and promoted myotube formation. Conversely, inhibition of miR-21-5p expression yielded opposing outcomes. These findings collectively confirm that miR-21-5p promotes C2C12 cell proliferation and differentiation by targeting *FBXO11*.

MiRNAs, including miR-34a and miR-324-5p, have been identified as key regulators of intramyocellular lipid deposition in myocytes [13,14]. Given that oleic acid significantly induces lipid deposition in C2C12 cells [26], miR-21-5p mimic and inhibitor, along with si-FBXO11, were transfected into oleic acid-treated C2C12 cells to assess their impact. Overexpression of miR-21-5p and silencing of *FBXO11* significantly reduced intramyocellular lipid accumulation and the expression of lipid-associated genes [43]. Conversely, inhibition of miR-21-5p expression resulted in increased lipid deposition and upregulation of lipid-associated genes. These findings demonstrate that miR-21-5p inhibits oleic acid-induced intramyocellular lipid deposition through the direct targeting of *FBXO11*. This observation aligns with previous reports suggesting that miR-21-5p mimics may serve as therapeutic agents to mitigate high-fat diet-induced weight gain in obese mice.

## CONCLUSION

This study identified miR-21-5p as a critical regulator of skeletal muscle development and intramyocellular lipid deposition in various skeletal pig breeds. miR-21-5p was found to enhance the proliferation and differentiation of myogenic cells while suppressing intramyocellular lipid accumulation by targeting the 3′UTR of *FBXO11*. These findings provide valuable theoretical insights into the molecular mechanisms underlying the variation in skeletal muscle formation among pigs with different meat quality, offering potential targets for improving meat production traits.

## SUPPLEMENTARY MATERIAL

**Supplement 1**. Primer information used in this study
