# Supplementary material for: MiR-21-5p enhances differentiation and mitigates oleic acid-induced lipid droplet accumulation in C2C12 myoblasts by targeting FBXO11
Source: Anim Biosci. 2025 Feb 27;38(6):1279–90. doi: 10.5713/ab.24.0665 (PMC12061579; doi:10.5713/ab.24.0665)
Supplement: Supplementary file 1 [file ab-24-0665-Supplementary-1.pdf]

Supplement 1. Primers used in this study

Primer name	Primer sequences (5′ to 3′)	Product Size (bp)
ssc-CDK4	F: AGTTTCTAAGCGGCCTGGAT<br>R: AACTTCAGGAGCTCGGTACC	188
ssc-CDK6	F: TAGCTGTCTCCACCACCCAC<br>R: GGCCATCTGTCGTTAGCCAG	122
ssc-PCNA	F: TAAAGAAGAGGAGGCGGTAA<br>R: TAAGTGTCCCATGTCAGCAA	175
ssc-CCND1	F: GCGTACCCTGACACCAATCTC<br>R: CTCCTCTTCGCACTTCTGCTC	183
ssc-MyoG	F: GCCCAGTGAATGCAACTCCCACA<br>R: CAGCCGCGAGCAAATGATCTCCT	98
ssc-MyoD	F: CAGGAACTGGGATATGGAGCT<br>R: TGAGTCGAAACACGGGTCAT	124
ssc-MyHC	F: CGCAAGAATGTTCTCAGGCT<br>R: GCCAGGTTGACATTGGATTG	110
ssc-FABP4	F: TGGGAACCTGGAAGCTTGTCTC<br>R: GAATTCCACGCCCAGTTTGA	197
ssc-FAS	F: CAGAAATCGCCTATGGTTGTT<br>R: ATTCAGGGTCATCCTGTCTCC	113
ssc-PPARγ	F: GGAAGACCACTCGCATTCCTT<br>R: GTAATCAGCAACCATTGGGTCA	121
ssc-CEBPα	F: AGCAACGAGTACCGGGTACG<br>R: TGTTTGGCTTTATCTCGGCTC	71
ssc-CEBPβ	F: ACTTCTACTACGAGCCCGACTG<br>R: TAGGGGCTGAAGTCGATGG	121
ssc-PBRM1	F: TGCATCTACACCGACAACTCCA<br>R:GTTGGAAATGAACTTCACGTCTGT	128
ssc-PDCD4	F: ACGGTGCTCCTGAGTATGTCTAA	153

	R: ATGTTCAGCTTCCGATATGTCTC	
ssc-FBXO11	F: TACAAAGAGCAATATGGAGGGTG<br>R: CACTTCATCTGGCAGTTTCTCC	151
ssc-GAPDH	F: TGGCAAAGTGGAGATTGTTGCC<br>R: AAGATGGTGATGGGCTTCCCG	156
mmu-FBXO11	R: CGTGTCCCACTATCAAGCATTG<br>R: CCTCAAAGCCTGCTATCCTGTT	264
mmu-GAPDH	F: TCGGAGTGAACGGATTTGGC<br>R: TGACAAGCTTCCCGTTCTCC	189
ssc-PBRM1	F: TGCATCTACACCGACAACTCCA<br>R: GTTGGAAATGAACTTCACGTCTGT	128
ssc-PDCD4	F: ACGGTGCTCCTGAGTATGTCTAA<br>R: ATGTTCAGCTTCCGATATGTCTC	153
ssc-miR-21-5p	F:CGCCGTAGCTTATCAGACTGATGTTGA<br>R: CAGTGCGTGTCGTGGAGT	
ssc-U6	F: CTCGCTTCGGCAGCACA<br>R: AACGCTTCACGAATTTGCG	
